# Influenza Pandemic Preparedness

**DOI:** 10.3201/eid0912.030289

**Published:** 2003-12

**Authors:** Kathleen F. Gensheimer, Martin I. Meltzer, Alicia S. Postema, Raymond A. Strikas

**Affiliations:** * Department of Human Services, Augusta, Maine, USA; † Centers for Disease Control and Prevention, Atlanta, Georgia, USA

**Keywords:** influenza, pandemic, preparedness, bioterrorism, resource allocation, priorities

In the list of potential bioterrorist agents, influenza would be classified as a category C agent (*1*). While previous influenza pandemics were naturally occurring events, an influenza pandemic could be started with an intentional release of a deliberately altered influenza strain. Even if a deliberately altered strain is not released, an influenza pandemic originating from natural origins will inevitably occur (*2*) and will likely cause substantial illness, death, social disruption, and widespread panic. Globally, the 1918 pandemic killed at least 20 million people (*3*). This figure is approximately double the number killed on the battlefields of Europe during World War I (*4*). In the United States alone, the next pandemic could cause an estimated 89,000–207,000 deaths, 314,000–734,000 hospitalizations, 18–42 million outpatient visits, and 20–47 million additional illnesses (*5*). These predictions equal or surpass many published casualty estimates for a bioterrorism event (*6*–*8*). In addition to the potential for a large number of casualties, a bioterrorism incident and an influenza pandemic have similarities that allow public health planners to simultaneously plan and prepare for both types of emergencies (Table).

**Table:** Planning for pandemic influenza and bioterrorism: similarities and differences^a,b^

**Issue**	Bioterrorist event	Pandemic influenza	
Likelihood	High	High	
Warning	None to days	Days to months	
Occurrence	Focal or multifocal	Nationwide	
Transmission/duration of exposure	Point source; limited; person-to-person	Person-to-person, 6–8 wks	
Casualties	Hundreds to thousands	Hundreds of thousands to millions	
First responders susceptible?	Yes	Yes	
Disaster medical team support/response	Yes	No (too widespread)	
Main site for preparedness, response, recovery, and mitigation	State and local areas	State and local areas	
*Essential preparedness components*			
Surveillance	Yes	Yes	
Law enforcement intelligence	Yes	No	
Investigation	Yes	Yes	
Research	Yes	Yes	
Liability programs	Yes	Yes	
Communication systems	Yes	Yes	
Medical triage and treatment plans	Yes	Yes	
Vaccine supply issues	Yes (for most likely threats)	Yes	
Drug supply issues	Yes	Yes	
Training/tabletop exercises	Yes	Yes	
Maintenance of essential community services	Yes	Yes	
*Essential response components*			
Rapid deployment teams	Yes	No	
Effective communications/media relations strategy	Yes	Yes	
Vaccine delivery	Yes (for some)	Yes	
Drug delivery	Yes (for most)	Yes	
Hospital/public health coordination	Yes	Yes	
Global assistance	Possibly	Yes	
Medical care	Yes	Yes	
Mental health support	Yes	Yes	
Mortuary services	Yes	Yes	
Supplies and equipment	Yes	Yes	
*Essential mitigation components*			
Enhanced surveillance	Yes	Yes	
Enhanced law enforcement intelligence	Yes	No	
Vaccine stockpile	Yes (selected agents)	Prototype vaccines only	
Drug stockpile	Yes	Yes	
Pre-event vaccination	Vaccination of selected groups^c^	Vaccination of groups at medical high risk with pneumococcal vaccine^d^	

^a^During a catastrophic infectious disease event, such as an influenza pandemic, there may be critical shortages of vaccines and drugs. Thus, clinics set up to administer vaccines and distribute antimicrobial drugs may require the services of a range of personnel whose fields of expertise are nonclinical. Examples of additional personnel that may be needed include law enforcement, translators, social workers, psychologists, and legal experts. ^b^Source: Adapted from: National Vaccine Program Office. Pandemic influenza: a planning guide for state and local officials (Draft 2.1). Atlanta: Centers for Disease Control and Prevention; 2000. ^c^At the time of writing, the smallpox vaccination program was just beginning. For other bioterrorist agents for which vaccines are available (e.g., anthrax), limited supplies and concerns about safety profiles have, up to this point, effectively prevented the widespread use of these vaccines. ^d^It may eventually be possible to vaccinate high-priority groups and the general population with a yet-to-be-developed “common epitope” vaccine, which might provide for a broader spectrum of protection against a variety of influenza A subtypes.

Preparing for both the next influenza pandemic and the next bioterrorist attack requires support and collaboration from multiple partners at the state, local, and federal level. Potential partners include the medical community, law enforcement, emergency management, and public health agencies. To help foster these crucial cross-discipline relationships, the Centers for Disease Control and Prevention (CDC) and the Council of State and Territorial Epidemiologists (CSTE), in collaboration with the National Emergency Management Association, the Association of State and Territorial Health Officials, the Federal Emergency Management Agency, and the Association of Public Health Laboratories, hosted a 2-day meeting on state and local pandemic influenza planning in May 2002. Over 125 officials representing epidemiology, communicable disease, laboratory, immunization, and emergency management programs from 46 states registered for this meeting. The objectives of the meeting were to enhance collaboration between state and local public health and emergency management agencies, establish mechanisms for integrating bioterrorism and pandemic influenza preparedness and response planning, and develop policy and strategy options for influenza pandemic preparedness and response at the state and local level. We report the results of a questionnaire distributed to the attendees; it was designed to elicit their views on the most important issues that must be addressed by a plan to respond to a catastrophic disease event.

## Priorities for Pandemic Influenza Planning

All plans for any catastrophic infectious disease event such as pandemic influenza or a bioterrorist attack must address five topics: surveillance and laboratory issues; communications; maintenance of community services; medical care; and supply and delivery of vaccines and drugs. After presentations providing background information, conference attendees were divided into breakout groups to discuss these topics. The groups did not discuss particular scenarios, but the presentations given before the breakout groups did include details of estimates of the potential impact of the next influenza pandemic (*5*). Attendees completed short (<5 questions), anonymous questionnaires at both the beginning and end of the breakout session. Each breakout group had a different set of questions relevant to the topic of that group.^1^ However, all groups addressed a common question, which asked persons to pick their top priority for a pandemic influenza response from one of the following options: reduce mortality, reduce morbidity, ensure continuation of essential services, reduce economic impact, and ensure equitable distribution of resources. As explained to the attendees before the breakout session, differences by age and risk group in rates of mortality and morbidity could mean that public health officials with limited resources might not be able to simultaneously maximize reductions in mortality and morbidity (*5*). The first three options were chosen most frequently (Figure). Even after discussion, no option was chosen by >50% of attendees, indicating that this group of professionals did not have a unified opinion regarding what the top priority should be to guide planning and response measures.

**Figure:**
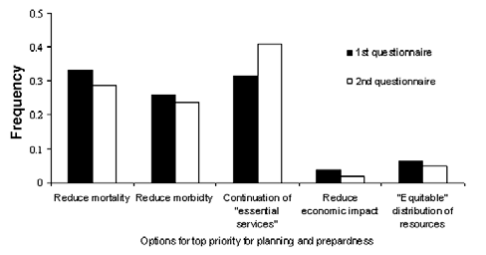
Distribution of responses identifying which goal should be the top priority for pandemic influenza planning and response (n = 107). During the conference, attendees were split into five groups for a breakout session. At the beginning and end of each such session, each attendee was given anonymous questionnaires. Each group had the same first question, in which attendees were asked to choose one of five options for top priority for influenza pandemic planning. This figure shows the frequency distribution of the attendees’ choices.

Conference attendees did, however, agree that global and domestic laboratory and disease surveillance must be strengthened to increase the likelihood of early detection and tracking of either pandemic influenza or a bioterrorist event. A rise beyond the baseline number of influenza-like illnesses (ILIs) could indicate a severe influenza season, arrival of pandemic influenza, or early warning of a bioterrorist attack with a pathogen that causes ILIs (e.g., anthrax). Thus, the number and accuracy of reports of ILI, ILI outbreaks, and laboratory-confirmed reports of influenza need to be increased. In addition, ensuring that adequate laboratory and disease surveillance systems are in place will benefit the public health response during yearly influenza epidemics. Conference attendees identified two critical gaps in infectious disease surveillance systems: 1) less than ideal or nonexistent systems to monitor outpatient and hospital-based ILI cases and 2) insufficient numbers of laboratory personnel and epidemiologists to monitor, provide diagnostic support, and respond to events.

Another critical component of any catastrophic infectious disease plan is communications. The anthrax attacks in 2001 demonstrated that the public, media, and healthcare professionals will demand accurate information, with frequent updates throughout the emergency. To minimize the potential for confusion, states and localities need to identify a recognized and trusted leader who will be the primary spokesperson to disseminate accurate information. Among attendees in the communications breakout group, 40% felt that the state governor would be the best spokesperson, 40% chose the state health officer, and 20% chose the state epidemiologist.

In the initial stages of, and potentially throughout, an influenza pandemic or a bioterrorist attack, there will be a shortage of many essential resources, including medical equipment and supplies, personnel, vaccines, and drugs. Prioritizing medical resources will therefore be necessary. The medical care breakout group unanimously chose state and local government as the authority that should prioritize and distribute healthcare resources. In the breakout group that discussed vaccine and antimicrobial agent issues, 73% chose essential workers and physicians as those who should be the first to receive vaccine and antiviral drugs. Only 27% chose those at high risk for adverse influenza-related health outcomes to be early recipients of vaccine.

## Conclusions

### Maximizing Resources and Planning Efforts

Conference attendees were well aware of the need to simultaneously plan and prepare for the next influenza pandemic and the next bioterrorist event. However, much work remains to be done. Without agreement regarding the top priority for allocating scarce resources, planning and implementing an optimal response to either pandemic influenza or a bioterrorist event will be difficult, if not impossible. Illustrating potential planning problems was the incongruity between the inability of most attendees to agree on the goal of planning and response measures (Figure) while 75% of a subgroup stated that essential workers and physicians should be the first to receive vaccines and antiviral drugs. In a situation with limited resources, usually only one goal can be optimized (either maximized or minimized) (*9*). Therefore, before accepting any of the initially limited supplies of vaccine and antiviral drugs, physicians and first responders will have to explain how such an allocation will help achieve the chosen top priority.

Unprecedented resources for enhancing the public health preparedness and response infrastructure have been recently provided to all states by congressional appropriations in the form of bioterrorism cooperative agreements. The request for proposals stated that planning moneys may be used “…to upgrade state and local public health jurisdictions’ preparedness for and response to bioterrorism, other outbreaks of infectious disease, and other public health threats and emergencies…” (*10*). Using such resources and reflecting upon the lessons learned from previous influenza pandemics and the 2001 terrorist events, public health, medical, and emergency management communities must work together to develop an effective plan to strengthen our national readiness to respond to any catastrophic infectious disease situation.

If our public health planning efforts are too narrowly focused on preparing responses to a few select bioterrorism-related scenarios, a new opportunity for planning responses to a broad spectrum of infectious disease-related catastrophes will be lost. Any plans made for responding to either pandemic influenza or bioterrorism events must include an explicit mechanism for making difficult decisions regarding the prioritization of scarce resources. The conference highlighted the need for all states to continue their discussions and public debates regarding the setting of priorities and methods for allocating scarce resources. Obviously, each state or local government will chose its own specific method for drawing up a plan to deal with catastrophic infectious disease events such as an influenza pandemic. To help aid the planning process, materials such as a planning guide are available from agencies such as CDC and CSTE. Ideally, such planning and prioritization activities should take place well in advance of any catastrophic infectious disease event.

## Footnotes

1 A complete copy of each questionnaire and a complete set of the results are available from the corresponding author.
